# Esophageal high-resolution manometry and 24 h pH-impedance monitoring normative values in patients with obesity candidate for bariatric and metabolic surgery

**DOI:** 10.1007/s13304-025-02167-4

**Published:** 2025-03-13

**Authors:** Salvatore Tolone, Edoardo Vincenzo Savarino, Nicola De Bortoli, Francesco Saverio Lucido, Claudio Gambardella, Luigi Brusciano, Simona Parisi, Gianmattia del Genio, Roberto Ruggiero, Ludovico Docimo

**Affiliations:** 1 General, Mininvasive, Oncologic and Bariatric Surgery Unit, Department of Advanced Medical and Surgical Sciences, University of Campania “Luigi Vanvitelli”, Via Pansini 5, 80131 Naples, Italy; 2 Division of Gastroenterology, Department of Translational Research and New Technologies in Medicine and Surgery, University of Pisa, Pisa, Italy; 3 Division of Gastroenterology, Department of Surgical, Oncological and Gastroenterological Sciences, University of Padua, Padua, Italy

**Keywords:** Bariatric surgery, GERD, High-resolution manometry, pH-impedance monitoring

## Abstract

Obesity is linked to increased risk of gastroesophageal reflux disease (GERD) and esophageal motility disorders, both of which may impact outcomes in metabolic and bariatric surgery (MBS). GERD pathophysiology in obesity includes elevated intraabdominal pressure and altered esophagogastric junction (EGJ) function. High resolution manometry (HRM) and 24-h esophageal pH-impedance (MII-pH) monitoring are vital in evaluating GERD, yet normative values specific to populations with obesity are limited, risking misdiagnosis if lean data are used. This study establishes normative HRM and MII-pH values in asymptomatic individuals with obesity, compared to normal-weight controls, to guide accurate diagnosis and treatment. A retrospective analysis was conducted on asymptomatic patients with obesity (BMI ≥ 30) and normal-weight controls (BMI 20–25) who underwent HRM and MII-pH prior to MBS between 2015 and 2024. Exclusion criteria included GERD symptoms, esophagitis, and prior gastrointestinal surgery. Key HRM parameters (LES pressure, EGJ morphology) and MII-pH metrics (acid exposure time, reflux episodes) were recorded and analyzed. Of the 96 patients with obesity and 25 normal-weight participants, significant differences in HRM and MII-pH results were observed. Individuals with obesity showed increased intra-gastric pressure, gastroesophageal pressure gradient, and higher acid exposure time. While LES pressure and EGJ morphology were similar to controls, participants with obesity exhibited distinct reflux patterns, especially postprandial, suggesting obesity-specific physiological changes. This study establishes normative HRM and MII-pH values for asymptomatic individuals with obesity, highlighting critical differences from normal-weight controls. Obesity-specific diagnostic criteria are essential for accurate GERD diagnosis, particularly for MBS candidates, to improve management and predict potential postoperative complications.

## Introduction

Obesity is a significant public health concern worldwide, with prevalence rates rising steadily [1]. Among different treatments, metabolic and bariatric surgery (MBS) is still the most effective option for achieving a considerable and durable weight loss [2]. Before undergoing BS, usually patients are checked for comorbidities that are commonly associated to obesity. It is well established that obesity is associated with an increased risk of gastroesophageal reflux disease (GERD) and other esophageal motility disorders [3]. The pathophysiology of GERD in individuals with obesity includes factors such as increased intra-abdominal pressure, altered esophagogastric junction (EGJ) function and anatomy, and changes in gastric emptying and in gut hormones levels [4]. Understanding the presence of GERD can be a crucial point, given the fact that BS can resolve or create a “de novo” reflux (especially after sleeve gastrectomy) [5]. However, only symptoms or endoscopic features usually are not sufficient to pone a correct diagnosis. Esophageal function tests such as high-resolution manometry (HRM) and 24-h esophageal pH-impedance monitoring (MII-pH) are crucial in the evaluation of esophageal motility disorders and GERD [6]. These tests provide detailed information about esophageal pressure dynamics and reflux patterns, enabling clinicians to diagnose conditions such as achalasia, distal esophageal spasm, and GERD with greater accuracy. Understanding normative values in different populations, particularly in individuals with obesity who are at higher risk for esophageal disorders, is essential for accurate diagnosis and treatment. Despite the clinical importance, there is a paucity of data on normative esophageal function values in populations with obesity, which complicates the diagnosis and management of esophageal disorders in these patients.

Previous studies have predominantly focused on normal weight individuals, and the normative values derived from these populations may not be applicable to patients with obesity [7–10]. This discrepancy highlights the need for population-specific normative data to ensure accurate diagnostic thresholds and effective treatment plans. For instance, the increased intra-abdominal pressure in individuals with obesity can affect the lower esophageal sphincter (LES) pressure and esophageal acid exposure, potentially leading to misinterpretation of HRM and pH-impedance results if normal weight normative values are used.

This study aims to fill this gap by establishing normative values for HRM and 24-h esophageal pH-impedance monitoring in asymptomatic individuals with obesity and comparing them with values from normal-weight controls. By doing so, we aim to provide a robust reference that can improve the diagnostic accuracy for esophageal disorders in the population with obesity. Additionally, understanding these differences can help tailor clinical interventions and management strategies, ultimately improving patient outcomes.

The primary objective of this study is to describe these normative values expressed in percentiles (5th, 25th, 75th, and 95th) for all common parameters used in HRM and pH-impedance monitoring. We will also compare these values to those of a cohort of normal-weight subjects to highlight any significant differences that may exist. This comparison will not only underscore the impact of obesity on esophageal function but also support the development of obesity-specific diagnostic criteria, which are crucial for the accurate assessment and management of esophageal disorders in this growing patient population.

## Materials and methods

This is a retrospective study from single center prospectively maintained database. All patients with obesity (body mass index, BMI, > 30) underwent symptomatic questionnaires (GERDQ score), upper endoscopy, HRM and MII-pH before surgery, as part of our internal protocol to choose for a restrictive procedure such as SG with or without fundoplication and hiatal hernia repair [11].

### Study design and patient selection

The study enrolled two cohorts: subjects with obesity (BMI ≥ 30) and normal-weight subjects (BMI 20–25). All participants were aged between 18 and 70 years, had no symptoms of reflux, and had a normal upper gastrointestinal endoscopy.

All patients who underwent different metabolic and bariatric procedures performed instrumental testing at our Bariatric surgical unit in Naples, Italy between 2015 and 2024. All participants provided written informed consent, and the study was approved by the institutional review board.

Inclusion criteria were: presence of class I or higher morbid obesity (defined as a body mass index, BMI, greater than 35 or higher than 30 in case of co-morbidities), indications to undergo MBS according to international and national guidelines [12–14], BMI 20–25 for normal-weight cohort; absence of typical (heartburn, regurgitation) and atypical GERD symptoms (such as dysphagia, non-cardiac chest pain, cough, etc.) as confirmed by clinical questionnaires; normal upper gastrointestinal endoscopy, with no evidence of any grade of esophagitis (according to the Los Angeles classification system grade A, B, C, D) or hiatal hernia (according to flap valve AFS classification) [15]; no history of gastrointestinal surgery; no use of proton pump inhibitors or other medications affecting esophageal motility in the past month.

Exclusion criteria were as follows: absence of any clinical or instrumental examination required by the study protocol, psychiatric illness, presence of reflux symptoms or other gastrointestinal symptoms, evidence of esophagitis or hiatal hernia on endoscopy, history of gastrointestinal surgery, use of proton pump inhibitors or other medications affecting esophageal motility in the past month, significant comorbidities that could affect esophageal function and declining consent to participate. Also, high pathological total acid exposure time in the esophagus and high pathological total number of refluxes events were considered as a further exclusion criteria after MII-pH-monitoring. The study was conducted according to the Helsinki declaration.

Assessment of anthropometrics (weight, height, BMI) was done in all subjects.

Typical reflux-related symptoms were investigated using the GERDQ score [16], a validated questionnaire incorporating a Likert visual analog scale (0–3, where 0 = absent, 1 = mild, 2 = moderate, and 3 = severe). Furthermore, atypical symptoms like cough, hoarseness, asthma, wheezing, and dysphonia were investigated by means of a 4-points Likert scale (0–3, where 0 = absent, 1 = mild, 2 = moderate, and 3 = severe). Then, patients underwent upper endoscopy, HRM and MII-pH.

### Pathophysiological esophageal evaluation

HRM and MII-pH were performed off medication (any antacid medication or prokinetic assumption was considered as an exclusion criteria), after an overnight fasting.

HRM was performed using a solid-state catheter (Given imaging, Los Angeles, CA, USA) with 36 circumferential pressure sensors spaced at 1-cm intervals. The catheter was inserted transnasally after an overnight fast and positioned to record from the hypopharynx to the stomach. Participants underwent a series of 10 swallows of 5 ml of water in the supine position. the following parameters were recorded: integrated relaxation pressure (IRP), distal contractile integral (DCI), distal latency (DL), peristaltic breaks, upper esophageal sphincter (UES) pressure, lower esophageal sphincter (LES) pressure, esophagogastric junction (EGJ) morphology, esophagogastric junction contractile integral (EGJ-CI), as previously described [17].

HRM data were also re-analyzed using the Chicago Classification version 4.0 criteria in other two highly expert Italian centers (in order to reduce bias analysis in a single center), adding the upright swallows and multiple rapid swallows when performed.

MII-pH was performed with a combined multichannel intraluminal impedance and pH catheter (Diversatek-Sandhill Scientific, Highlands Ranch, Co, USA). The catheter was placed transnasally, with impedance sensors positioned at 3, 5, 7, 9, 15, and 17 cm above the LES and pH sensors 5 cm above the LES. Participants maintained their usual activities and diet, avoiding acidic foods and beverages. They recorded their symptoms, meals, and body position changes in a diary. The following parameters were analyzed: total acid exposure time (%, AET), acid clearance time (total, in seconds), DeMeester score, symptom association probability (SAP), total number of reflux episodes, number of acid reflux events, number of weakly acid reflux events, number of weakly alkaline reflux events, mean nocturnal baseline impedance levels, number of proximal reflux events [18].

### Statistical analysis

Statistical analysis was performed using SPSS for MAC OSX (version 22; SPSS inc., Chicago, Il, USA). Continuous data are expressed in percentiles (5th, 25th, 75th, and 95th), unless otherwise stated. The Wilcoxon signed-rank test for paired data was used for comparison of means in the same patients pre- and post-operatively. Unpaired t test was used for comparison of means in different procedures. A two-sided p-value of 0.05 was considered statistically significant.

## Results

During the study period, 426 subjects with obesity (289 females, 37 ± 16 years-old, mean weight 131 (95–202) kg, mean BMI 43 (30–81) kg/m^2^ were studied. Among these, 148 were asymptomatic, with a normal upper endoscopy. Of these, 108 underwent to HRM and MII-pH; 12 had a strong pathological AET and/or total number of refluxes, so they were excluded from the study. The remaining 96 subjects constituted the normative obesity cohort, whereas 25 asymptomatic normal weight subjects (13 females, 22 ± 4 years-old, mean weight 69 (52–91) kg, mean BMI 22 (20–24) kg/m^2^, non-smokers, none with co-morbidities) who previously underwent HRM and MII-pH in our lab constituted the normative lean control cohort [19].

### HRM findings

Mean LES pressure was 28.4 +–3.5 mmHg (5th 10.1–95th 55.3), hiatal hernia was present in three subjects (EGJ type II, mean size 0.9 cm). Mean percent of intact peristalsis was 88%, with a mean DCI of 2656 +–376 (5th 456–95th 5976). Pathological HRM trace were present in six patients (6.2%); one patient had EGJ outflow obstruction, two had hypercontractile esophagus and three had ineffective motility. Complete HRM data are depicted in Table 1.

**Table 1:** High-resolution manometry complete findings (mean, 5th, 25th, 75th and 95th percentile) in Obese and in lean controls populations

HRM	Obese					Lean controls				
	Mean	5th percentile	25th percentile	75th percentile	95th percentile	Mean	5th percentile	25th percentile	75th percentile	95th percentile
Supine swallows variables										
EGJ resting pressure (mmHg)	28,4	10,1	21,3	32,4	55,3	32	11	28	39	44
EGJ IRP (mmHg, median)	10,6	2	5,5	14,2	17	8	0	2	6	13
EGJ-CI (mmHg*cm)	24	6	20	28	55	35	8	30	40	56
IGP (mmHg)	15,2	7,4	13	18,2	21	6	3	5	7	11
GEPG (mmHg)	11,2	5,1	9,1	14	16,5	5	3	3	7	9
Hiatal hernia size (cm)	0	0	0	0,2	0,9	0	0	0	0	0,2
DCI (mmHg*cm*sec)	1949	499	1423	2381	5485	2453	525	1568	2866,3	5889
DL (sec)	6,2	5,1	5,7	7,5	7,9	6,3	4,9	5,6	7,5	7,7
Intact swallows (%)	85	60	80	100	100	90	70	80	100	100
Weak swallows (%)	9,3	0	0	20	40	8	0	0	20	30
Failed swallows (%)	2,5	0	0	10	20	1	0	0	0	10
Fragmented swallows (%)	1	0	0	0	10	1	0	0	0	10
Premature swallows (%)	1	0	0	0	10	0	0	0	0	0
Hyper-contractile swallows (%)	1	0	0	0	20	0	0	0	0	10
Panpressurization (%)	0,2	0	0	0	10	0	0	0	0	0
**Upright swallows variables**										
EGJ resting pressure (mmHg)	19,4	4	12,5	22,1	29,7	18,3	9	15,6	22,5	28,6
EGJ IRP (mmHg, median)	8,1	2,2	3,4	13,2	13,4	6	0	1	4	8,9
IGP (mmHg)	11	5,3	8,2	13,8	17	5	2	4	6	10
GEPG (mmHg)	9,2	4,6	7,1	12,5	15	4	2	2,5	5,3	6,8
Hiatal Hernia size (cm)	0	0	0	0,2	0,9	0	0	0	0	0,2
Intact swallows (%)	72	50	60	100	100	75	0	50	100	100
Weak swallows (%)	21	0	0	30	50	14	0	0	30	40
Failed swallows (%)	5	0	0	20	30	7	0	0	10	20
Fragmented swallows (%)	0,5	0	0	0	12	4	0	0	10	10
Premature swallows (%)	0,5	0	0	0	12	0	0	0	0	0
Hyper-contractile swallows (%)	1	0	0	12	25	0	0	0	0	0
Panpressurization (%)	0	0	0	0	0	0	0	0	0	0

*HRM* high-resolution manometry, *EGJ* esophagogastric junction, *IRP* integral relaxation pressure, *EGJ-CI* esophagogastric junction contractile integral, *IGP* intragastric pressure, *GEPG* gastroesophageal pressure gradient, *DCI* distal contractile integral, *DL* distal latency

### MII-pH findings

Mean total AET% was 2.9 +–0.7 (5th 0.2–4.5 95th), whereas mean total reflux numbers was 32 +–12 (5th 13–95th 68). Regarding chemical quality, acid refluxes were more represented than weakly acid and weakly alkaline (74.4%, 25.5% and 0.1%, respectively). Complete MII-pH data are shown in Table 2.

**Table 2:** Multichannel intraluminal impedance pH-monitoring complete findings (mean, 5th, 25th, 75th and 95th percentile) in Obese and in lean controls populations

	Obese					Lean controls				
	Mean	5th percentile	25th percentile	75th percentile	95th percentile	Mean	5th percentile	25th percentile	75th percentile	95th percentile
MII-pH monitoring variables										
**AET % pH < 4**										
Total	2,9	0,2	1,2	3,6	4,5	1,8	0	0,7	2,8	4,1
Upright	3,2	0,2	1,2	3,8	4,7	2,1	0,3	1,1	3,1	4,2
Recumbent	0,7	0	0,4	1,1	1,4	0,2	0	0	0,5	0,8
**ACT (sec)**	67	19	45	110	122	64	20	43	102	118
**DeMeester Score**	14	3	5	15	18	10	0	2	12	14
**Number of reflux events at MII**										
Total	35	6	26	46	62	24	4	14	34	41
Upright	30	5	22	40	51	22	4	11	31	37
Recumbent	10	1	4	12	14	5	0	3	8	9
**Acid reflux pattern at MII**										
Total	26	3	15	38	53	19	2	9	26	34
Upright	27	4	13	37	49	17	2	8	25	31
Recumbent	5	1	1	11	12	3	0	1	5	7
**Weakly Acid reflux pattern at MII**										
Total	9	1	5	15	17	6	0	2	8	11
Upright	5	1	4	10	13	5	0	2	8	10
Recumbent	4	0	1	5	8	1	0	0	3	4
**Weakly Alkaline reflux pattern at MII**										
Total	0	0	0	3	4	0	0	0	1	1
Upright	0	0	0	3	4	0	0	0	1	1
Recumbent	0	0	0	1	1	0	0	0	0	1
**Post Prandial reflux events at MII**	29	17	22	34	38	10	3	6	12	16
**MNBI (Ohms)**	2786	2122	2324	2891	3011	2845	2231	2513	2996	3125

*MII* multichannel intraluminal impedance, *AET* acid exposure time, *ACT* acid clearance time, *MNBI* mean nocturnal baseline impedance

### Comparison with lean controls

Many mean data from HRM and MII-pH were similar in patients with obesity and in lean controls. A statistically significant difference was recorded for total AET% (both for mean and 95th values, *p* < 0.05) and for total number of reflux episodes (both for mean and 95th values, *p* < 0.05).

## Discussion

Obesity is demonstrated to impair the esophageal function [3]. The previous studies about normative values for HRM and MII-pH were performed in lean patients, so the limit range proposed may not be applicable to a patient with obesity [7–10]. In this study, we aimed to describe normative values in an accurately selected cohort of patients with obesity before undergoing MBS. We found that HRM values were similar to those in lean controls except for mean Intragastric pressure (IGP), consequently the gastroesophageal pressure gradient (GEPG), and the 95th IRP data. This latter resulted to be higher (median 17) than the conventional limit of 15 that is currently used in the Chicago Classification v4.0 for the definition of the impaired relaxation of the EGJ. Given this “new” evidence, the cut-off for this definition in a patient with BMI > 30 should be raised to 17. On the other hand, we found any achalasia and only one EGJ-OO, confirmed with the upright swallow protocol, that is essential in these setting of patients because of the probability of having a higher IRP. Furthermore, the esophageal motility was really similar to the lean controls. The most common motility pattern was represented by intact peristalsis, followed by a low percentage of weak and failed peristaltic swallows and episodical hypercontractile, panesophageal and premature contractions. Perhaps, this pattern we found is similar to a systematic review by Alcala et al. [20], and the multicentric study by Rogers et al. [7]. Our results seem to be in contrast with previous papers in which there were several reported dysmotility (especially hypercontractility, and ineffective peristalsis, as well as a disrupted barrier function of EGJ with increased incidence of hiatal hernia). This discrepancy is probably due because the aforementioned studies were performed in obesity cohorts without any stratification regarding the presence of symptoms or a disrupted anatomy. As for MII-pH, this is to our knowledge, the largest normative cohort study up to date. We found a statistically significant difference with lean controls regarding mean total AET, 95th total AET, the mean total number of reflux episodes detected at MII, and the number of post-prandial refluxes. The percentage distribution of reflux chemical quality (i.e. acid, weakly acid and weakly alkaline) was similar in the two cohort. Different dataset for normative values for MII-pH off therapy exist, from single center to multicentric ones [9, 10, 21, 22]. The upper (95th) limit of AET in subjects with obesity seems to be higher than other dataset, as well as the total number of reflux events. According to the recent Lyon Consensus V2.0 for GERD diagnosis [6], these values would fall into the so called “grey area” in which adjunctive parameters are needed in order to confirm GERD presence. Also, it seems that the increased acid exposure is linked to an increased number of total reflux episodes, more frequent in the post-prandial period. Thus, it could be speculated that this increased acid pattern is caused by an increased ingested volume of food during meals or by the increased number of meals (or snack), as well as the increased consumption of junk food, carbonated beverages and all the other foods that can impair the EGJ barrier. According to the present results, it is of note to understand if HRM and pH-monitoring should be performed in all subjects before undergoing MBS. Up-to-date, only the implementation of esophageal instrumental testing can give an objective evaluation of GERD presence; on the other hand, routine performance can be challenging, because of not all MBS centers are equipped for esophageal study, and because it is still not demonstrated to be a cost-effective strategy. Thus, we believe that the future research should focus on how we could select MBS patients and testing them effectively for GERD.

In the present study, one of the major points of strength is the accurate selection of both asymptomatic and with normal EGJ anatomy, without any sign of esophagitis or abnormal acid exposure in esophagus. Thus, we aimed to select an ideal normative cohort of patients with obesity that could have result in an optimal data set of real normative values for HRM and MII-pH, because only using symptom assessment even with conventional scoring system (as the GERDQ) can be biased by an high percentage of patient with obesity that are affected by silent reflux [23]. One limitation of our study in this case is represented by the choice of not imposing a standardized meal, as performed in the study by Savarino et al. with a Mediterranean diet [24]; this could have led to a heterogeneous reflux pattern, but we preferred to determine reflux exposure in the obesity cohort without interfering with their daily habits. Another possible explanation can lie in the increased IGP and GEPG we found at HRM. The increased IGP caused by the increased abdominal visceral fat creates a pressure gradient toward the negative pressure zone into the thoracic esophagus (namely, the GEPG), allowing the possibility of increased transient LES relaxations and consequently, increasing the reflux episodes [19, 25]. Our results are also different from the normative value cohort presented by Doulami et al. [22], in which total AET values were higher than those in our cohort. We could speculate that this difference can be interpreted by the disparity of the numerosity in the two cohorts (25 in Doulami’s paper and 96 in the present study) and by the fact we cropped frankly pathological MII-pH tracings from the final analysis because we identified them as “silent reflux”, whereas those pattern were included in Doulami’s study.

## Conclusions

This is a preliminary study which lay the foundations for normative values for HRM and 24-h esophageal pH-impedance monitoring in asymptomatic and endoscopy-negative individuals with obesity, compared to normal-weight controls. These values are crucial for the accurate diagnosis of esophageal disorders in the population with obesity. The significant differences observed underscore the need for obesity-specific diagnostic criteria in esophageal function testing, that can be used in future as baseline especially when compared to those underwent metabolic and bariatric surgery and that can develop esophageal complications.
